# Increased mean perfusion pressure variability is associated with subsequent deterioration of renal function in critically ill patients with central venous pressure monitoring: a retrospective observational study

**DOI:** 10.1080/0886022X.2022.2120822

**Published:** 2022-11-11

**Authors:** Yudie Peng, Buyun Wu, Changying Xing, Huijuan Mao

**Affiliations:** Department of Nephrology, The First Affiliated Hospital of Nanjing Medical University, Jiangsu Province Hospital, Nanjing, China

**Keywords:** acute kidney injury, critical illness, mean perfusion pressure, renal hemodynamics, variability

## Abstract

**Purpose:**

The mean perfusion pressure (MPP) was recently proposed to personalized management tissue perfusion pressure in critically ill patients. Increased MPP variability (MPPV) may be associated with organ injuries. Our objective was to determine if increased MPPV was associated with subsequent deterioration of renal function in critically ill patients.

**Methods:**

We analyzed data stored in the eICU-CRD and MIMIC-IV databases. The exposure was MPPV, measured as the coefficient of variation (CV) using the MPP data of the first 24 h after first ICU admission. The primary endpoint was deterioration of renal function, defined as new-onset or progress of acute kidney injury between 24 and 72 h after ICU admission.

**Results:**

The study population consisted of 8,590 patients from eICU-CRD and 6,723 patients from MIMIC-IV database. A total of 28.4% and 30.2% of the study population experienced deteriorated renal function, respectively. Patients with deteriorated renal function had significantly higher median MPP-CV compared with those without (12.2% vs 11.5% and 12.8% vs 12.5%, *p* < .001). In fully adjusted multivariate logistic models, higher MPP-CV (adjusted OR per 1-SD, 1.08; 95% CI, 1.02–1.13 and adjusted OR per 1-SD, 1.06; 95% CI, 1.00–1.12, respectively) was significantly associated with greater risk of primary endpoint. The pooled analyses showed heterogeneity in patients with cardiac surgery, medical sepsis and others.

**Conclusion:**

Increased MPPV was associated with an increased risk of subsequent deterioration of renal function in critically ill patients with central venous pressure monitoring. Maintaining stable MPP may reduce the risk of renal function deterioration.

## Background

Blood pressure variability (BPV) is a parameter that characterizes the continuous dynamic fluctuation of blood pressure (BP) during a specific period [1]. The fluctuations can be caused by the external environment and psychological factors, internal neurohumoral regulation, and their complex interactions. Although these mechanisms have not been thoroughly studied [2], these complex body regulations make organ perfusion reach dynamic balance and maintain homeostasis. Recent studies have shown that long-term abnormal BPV is related to renal damage [3–5], high risk of cardiovascular and cerebrovascular events, and mortality, independent of the average BP level [6–9].

The microvascular system of the kidney is most vulnerable to the adverse effects of larger BPV which is associated with higher albuminuria [10]. In healthy people, the kidney can maintain its perfusion through tubuloglomerular feedback when mean arterial pressure (MAP) fluctuates in the range of 80 ∼ 180mmHg [11]. However, the physiological regulation function is impaired in critically ill patients. Affected by factors like sepsis, nephrotoxin, and insufficient blood perfusion [12–14], abnormal BPV may be a hit to the kidney and finally deteriorate renal function. Previous studies have rarely focused on the population of critically ill patients. One study conducted by Xie and his colleagues has shown that in critically ill patients, higher systolic BPV was related to the incidence of acute kidney injury (AKI) [15], which is a clinical syndrome characterized by a significant acute reduction in glomerular filtration rate and occurs in more than 50% of patients admitted to intensive care units [16]. However, the sample size of this study was relatively small and had no external verification. In addition, they also did not distinguish between the time of BPV monitoring and the time of AKI occurrence. Moreover, traditional index like systolic blood pressure (SBP) and diastolic blood pressure (DBP) has some physiological deficiencies, especially the failure to consider venous outflow pressure. Obtained by the difference between MAP and central venous pressure (CVP), mean perfusion pressure (MPP) was recently proposed to personalized management tissue perfusion pressure instead of MAP [17,18].

Accordingly, we sought to explore the relationship between BPV, to be exact, MPP variability (MPPV) and the subsequent deterioration of renal function in critically ill patients. We hypothesized that increased MPPV might be associated with deterioration of renal function afterward.

## Methods

### Study population

All patients in the eICU Collaborative Research Database (eICU-CRD) version v2.0 [19], and Medical Information Mart for Intensive Care (MIMIC)-IV version 0.4 [20] were eligible for the inclusion. The inclusion criteria were [1] age 16 years or more and [2] at least 12 h of continuous MAP and CVP invasive monitoring within the first 24 h in the first ICU stay. Those who received dialysis, died during the first 24 h, complicated with chronic kidney disease (CKD) stage 5, diagnosed with acute compartment syndrome, or had incomplete data (missing data in the Charlson comorbidity index, stage of AKI or mortality) or underwent nephrectomy or low-quality MPP data (defined as the number of recorded MPP value less than 12 or the monitoring interval of MPP larger than 3 h) were excluded.

### Data extraction and definitions

We extracted all variables through Structured Query Language, including time-weighted average MPP (TWA-MPP), demographic data, baseline ICU characteristics, Charlson comorbidity index [21], the modified Sequential Organ Failure Assessment (SOFA) (SOFA score minus cardiovascular component which was collected separately) [22], Oxford Acute Severity of Illness Score (OASIS) [23] and the incidence of AKI. Vital signs were archived into the database as 5-min median values in eICU-CRD database and 60-min values in MIMIC-IV database. MAP and CVP were monitored at the same minute. The method for evaluating OASIS is displayed in Supplementary figure 1.

Sepsis was defined using the third sepsis definition, that is, patients with suspected infection with associated organ dysfunction (SOFA ≥2) [24,25]. The suspected infection was defined as the acquisition of a body fluid culture temporally contiguous to the administration of antibiotics. AKI was defined using serum creatinine and urine volume according to the KDIGO guidelines of AKI [26], in which the stage of AKI was defined as the higher stage in creatinine standard and urine output standard. The baseline serum creatinine was assessed using the following method: if the estimated glomerular filtration rate (eGFR) based on the lowest creatinine in the past 7 days was >75 mL/min per 1.73 m^2^, we used the lowest creatinine throughout the past 7 days. If eGFR based on the lowest creatinine in the past 7 days was not available or < 75 mL/min per 1.73 m^2^, we used the serum creatinine calculated back from the MDRD equation set to a standard GFR of 75 mL/min per 1.73 m^2^.

### Exposure

Short-term MPPV was measured as the coefficient of variation (CV) of MPP during the first 24 h of ICU stay. MPP was calculated as MAP minus CVP.

### Endpoints

The primary endpoint was the deterioration of renal function, which was defined as new-onset or progress of AKI between 24 and 72 h following the ICU admission. New-onset of AKI was defined as AKI identified between 24 and 72 h of ICU stay in those patients without AKI recognized within the first 24 h. The progress of AKI was defined as the stage of AKI increase in the subsequent 48 h [27].

### Statistical analysis

The categorical variables were presented as counts and percentages, including 95% confidence intervals (CI) where applicable, and continuous variables as medians and interquartile ranges (IQR) as the distributions are skewed. According to the analyzed cohort, patients were categorized into groups, including eICU-CRD and MIMIC-IV.

The association between MPP-CV and the endpoints were plotted using general additive models firstly, which allowed for parametric and nonparametric forms of relationship between a continuous predictor and a continuous, normally distributed or non-normally distributed outcome [28]. We then used univariate (model 1) and multivariate (models 2, 3, 4 and 5) logistic regression to determine the standard odds ratio (sOR) and 95% CI between MPP-CV and the subsequent deterioration of renal function. In these models, MPP-CV was taken as a continuous variable normalized to 1 SD to allow for comparing the intensity of correlation across databases. Model 2 was adjusted for demographic covariates including age, gender, body mass index (BMI), and ethnicity. Model 3 was further adjusted by surgery admission, cardiovascular ICU, history of hypertension, history of diabetes, history of CKD, history of chronic heart failure (CHF), modified SOFA score and OASIS during the first 24 h. Model 4 was additionally adjusted by cumulative vasopressor dose during the first 24 h, sedatives use or not during the first 24 h, mechanical ventilation or not during the first 24 h, transfusion of RBCs during the first 24 h, AKI at the first 24 h, sepsis or not before the 24 h of ICU admission, exposure to ≥2 nephrotoxins before and after 24 h of ICU admission compared with Model 3. Model 5 was further adjusted by time-weighted average MPP (TWA-MPP) based on Model 4. TWA-MPP during the first 24 h of ICU stay was calculated as the area under the MPP–versus–time plot as follows:

$$\text{TWA}\;=\;\left[\left(t_{2}-t_{1}\right)\left(X_{1}+X_{2}\right)/2+\left(t_{3}-t_{2}\right)\left(X_{2}+X_{3}\right)/2+\ldots +\left(t_{n}-t_{n-1}\right)\left(X_{n-1}+X_{n}\right)/2\right]/\left(t_{n}-t_{1}\right)$$



where X_n_ is the value of the variable of interest at the timepoint t_n_.

Moreover, we varied the robustness of our study through several secondary analyses. First, we took variation independent of the mean (VIM) [29] as another exposure of interest. VIM is a variability measurement which has no correlation with mean BP levels and detailed formula was displayed in Supplementary table 1. VIM is based on the MPP distribution within each cohort, so the value itself cannot be compared across populations. Second, baseline creatinine determined by the nadir creatine during seven days before and after ICU admission was used to confirm the association between MPP-CV of the first 24 h of ICU stay and the subsequent deterioration of renal function. Third, to evaluate the consistency of our research among different populations, we conducted subgroup analyses on the primary endpoint in the pooled population. Finally, we conducted the pooled analysis in the cardiac surgery patients, medical septic patient and others using univariate (model 1) and multivariate (models 2, 3, 4 and 5) logistic regression to determine the relationship between MPP-CV and the endpoint. All the analyses were performed with R version 4.0.3 software, and a two-sided *p* < .05 was considered statistically significant.

## Results

### Patient characteristics

**Figure 1.**
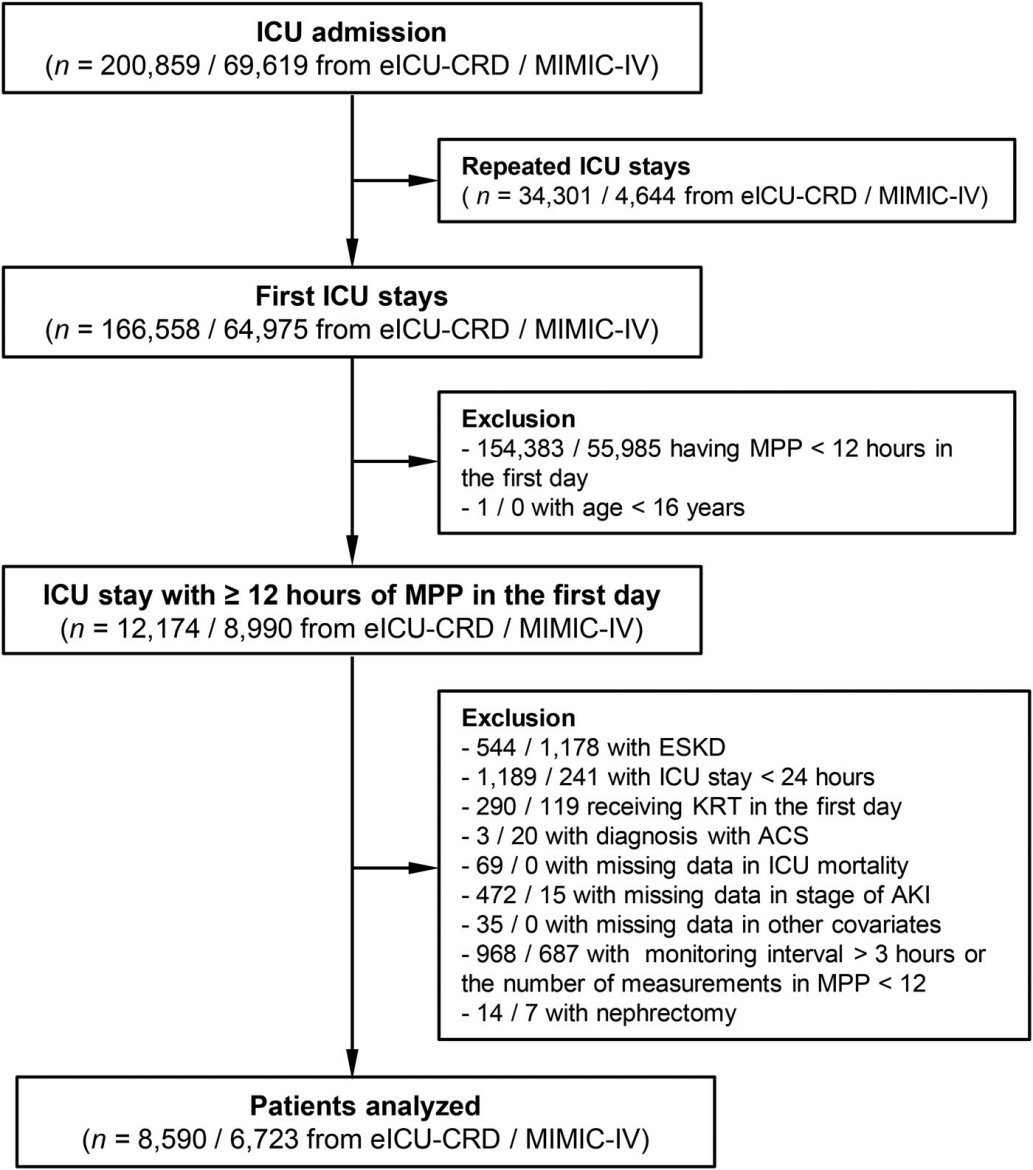
Patient flow chart.

The study population consisted of 8,590 patients admitted from 2014 to 2015 in the eICU database and 6,723 patients admitted from 2008 to 2019 in the MIMIC-IV database who met all the inclusion and exclusion criteria (Figure 1). Baseline characteristics and BPV of the study population between with or without deterioration of renal function are summarized in Table 1.

**Table 1.** Baseline characteristics between with or without deterioration of renal function in the next 48 h.

	eICU-CRD (*N* = 8590)			MIMIC-IV (*N* = 6723)		
Variables	No deterioration of renal function (*N* = 6153)	Deterioration of renal function (*N* = 2437)	*p* value	No deterioration of renal function (*N* = 4695)	Deterioration of renal function (*N* = 2028)	*p* value
Age (year)	66 (57, 75)	69 (60, 77)	<.001	68 (60, 75)	69 (61, 77)	<.001
Male (%)	3940 (64.0)	1524 (62.5)	.20	3263 (69.5)	1376 (67.9)	.19
Body mass index (kg/m^2^)	28.4 (24.7, 33.2)	29.0 (25.1, 33.6)	.004	28.4 (25.1, 32.6)	27.6 (24.4, 31.4)	<.001
White race (%)	4812 (78.2)	1951 (80.1)	.06	3469 (73.9)	1463 (72.1)	.14
Surgical admission (%)	4708 (76.5)	1853 (76.0)	.66	3353 (71.4)	1421 (70.1)	.28
Cardiovascular ICU (%)	3240 (52.7)	1367 (56.1)	.004	4271 (90.0)	1849 (91.2)	.82
History of hypertension (%)	3673 (59.7)	1478 (60.6)	.43	3544 (75.5)	1474 (72.7)	.02
History of diabetes (%)	1883 (30.6)	870 (35.7)	<.001	1059 (22.6)	419 (20.7)	.09
History of CKD (%)	686 (11.1)	293 (12.0)	.27	740 (15.8)	386 (19.0)	.001
History of CHF (%)	990 (16.1)	449 (18.4)	.01	1156 (24.6)	506 (25.0)	.80
Vasopressor dose (NE, mg)	0.0 (0.0, 1.6)	0.0 (0.0, 4.7)	<.001	0.9 (0.06, 4.2)	1.8 (0.2, 6.4)	<.001
Vasopressor use (%)	3111 (50.6)	1423 (58.4)	<.001	3724 (79.3)	1694 (83.5)	<.001
Sedatives (%)	2586 (42.0)	1136 (46.6)	<.001	3759 (80.1)	1691 (83.4)	.002
Mechanical ventilation (%)	4436 (72.1)	1980 (81.2)	<.001	4540 (96.7)	1979 (97.6)	.06
Transfusion of RBCs (mL)	0 (0, 0)	0 (0, 0)	.21	0 (0, 0)	0 (0, 3.5)	<.001
Transfusion of RBCs (%)	327 (5.3)	147 (6.0)	.23	1106 (23.6)	590 (29.1)	<.001
Sepsis (%)	2185 (35.5)	1018 (41.8)	<.001	4092 (87.2)	1775 (87.5)	.71
Exposure t*o* ≥ 2 nephrotoxins (%)	1019 (16.6)	484 (19.9)	<.001	1384 (29.5)	609 (30.0)	.67
AKI in the first 24 h (%)	2772 (45.1)	1239 (50.8)	<.001	3550 (75.6)	867 (42.8)	<.001
Modified SOFA Score	6 (4, 8)	7 (5, 9)	<.001	4 (2, 6)	4 (3, 6)	<.001
OASIS	29 (23, 35)	31 (25, 37)	<.001	34 (29, 39)	34 (30, 39)	.14
TWA-MAP (mmHg)	74.6 (69.8, 80.4)	73.1 (68.2, 79.0)	<.001	73.6 (69.8, 77.6)	73.5 (69.7, 77.2)	.50
TWA-CVP (mmHg)	10.3 (7.6, 13.1)	11.1 (8.5, 14.2)	<.001	9.4 (6.9, 12.2)	9.8 (7.3, 12.6)	<.001
TWA-MPP (mmHg)	64.0 (58.8, 70.4)	61.8 (56.2, 68.3)	<.001	63.9 (59.4, 68.7)	63.4 (58.9, 68.1)	.014
MPP-CV (%)	11.5 (9.3, 14.4)	12.2 (9.7, 15.0)	<.001	12.5 (10.1, 15.3)	12.8 (10.3, 16.0)	.001
MPP-VIM (units)	0.19 (0.16, 0.24)	0.20 (0.16, 0.25)	<.001	0.80 (0.65, 0.98)	0.82 (0.67, 1.02)	.002

Continuous variables were expressed as median (interquartile range) as the distributions are skewed and categorical variables were expressed as number (percentage).

AKI: acute kidney injury; CHF: chronic heart failure; CKD: chronic kidney disease; CVP: central venous pressure; ICU: intensive care unit; MAP: mean arterial pressure; MPP: mean perfusion pressure; NE: norepinephrine equivalents; Oxford Acute Severity of Illness Score; SOFA: Sequential Organ Failure Assessment; TWA: time weighted-average.

The majority of patients in the two cohorts were admitted to the hospital due to surgery, in which cardiovascular ICU accounted for a large proportion in both cohorts. More hospital information, exposure data, and endpoints are presented in supplementary Table 2.

### Exposures and endpoints

Of the study population in the two databases, the median MPP-CV (IQR) in eICU was 11.7% (9.4%, 14.6%) and 12.6% (10.2%, 15.5%) in MIMIC-IV (supplementary Table 2). There were 28.4% in eICU and 30.2% in MIMIC-IV identified deterioration of renal function within 24 to 72 h after admission to ICU, respectively. The patients with deterioration of renal function had significantly higher MPP-CV [eICU: 12.2% (9.7%, 15.0%) versus 11.5% (9.3%, 14.4%), *p* < .001; MIMIC-IV: 12.8% (10.3%, 16.0%) versus 12.5% (10.1%, 15.3%), *p* = .001] and MPP-VIM levels [eICU: 0.20 (0.16, 0.25) versus 0.19 (0.16, 0.24), *p* < .001; MIMIC-IV: 0.82 (0.67, 1.02) versus 0.80 (0.65, 0.98), *p* = .002] as compared to those without deterioration of renal function (Table 1).

### Association between MPP-CV and deterioration of renal function

**Figure 2.**
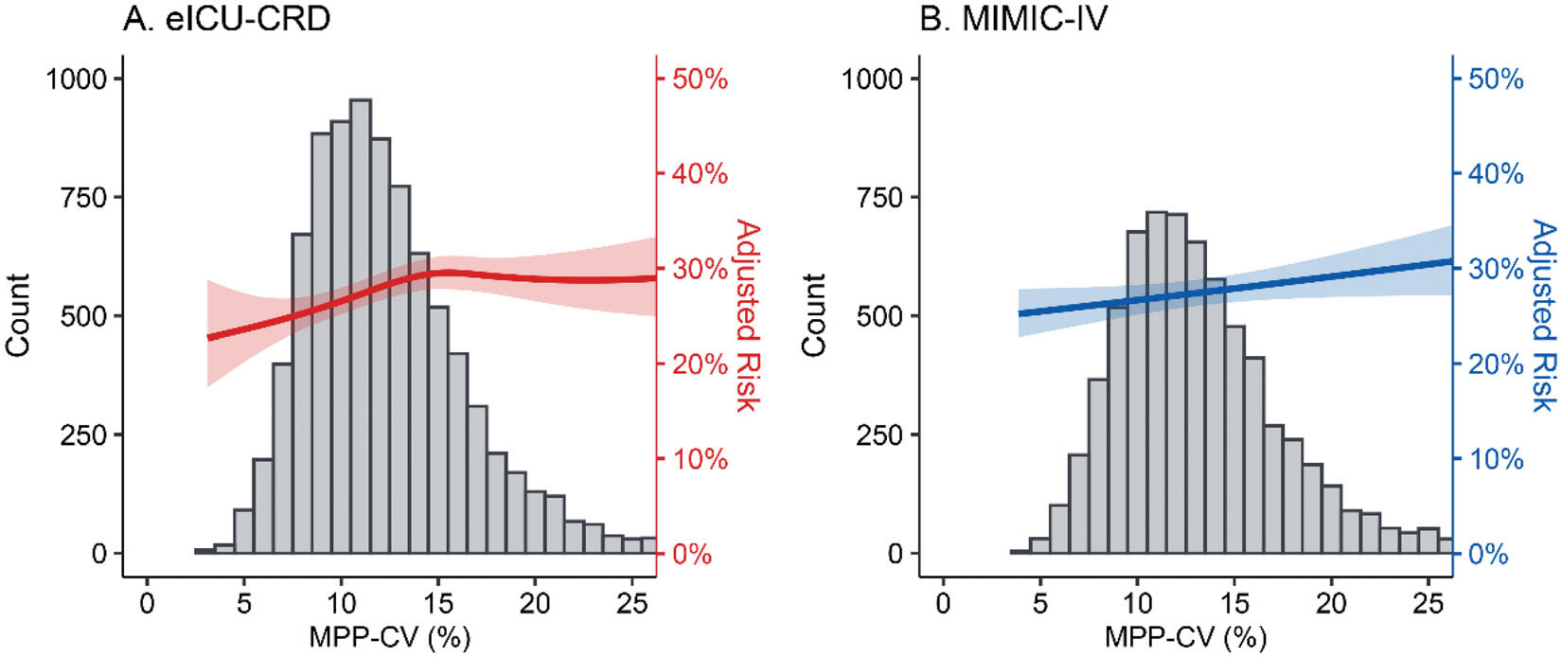
The association between MPP-CV and subsequent deterioration of renal function fitted by general additive models and the histograms of MPP-CV in two databases.

The risk of increased MPP-CV and deterioration of renal function within 24 to 72 h after ICU admission showed a significant positive relationship no matter in univariate (model 1) and multivariate logistic regression models (models 2, 3, 4, and 5) in both databases (Table 2). The unadjusted sOR of deterioration of renal function for per 1-SD increase in MPP-CV was 1.13 (95% CI, 1.08–1.18, *p* < .001) in the eICU and 1.10 (95% CI, 1.04–1.16, *p* < .001) in the MIMIC-IV database, respectively. Moreover, the findings remained significant in four multivariate logistic regression models after adjusting for baseline demographic data, comorbidities, admission illness severity scores, risk factors for AKI, and TWA-MPP in succession. In the fully adjusted model, model 5, the adjusted sOR for deterioration of renal function was 1.08 (95% CI, 1.02–1.13, *p* = .003) and 1.06 (95% CI, 1.00–1.12, *p* = .05). The relationship between the incidence of deterioration of renal function and MPP-CV accounting for all confounders was shown in Figure 2.

**Table 2.** Association of MPPV and subsequent deterioration of renal function in two cohorts.

Models for subsequent deterioration of renal function	eICU-CRD		MIMIC-IV	
	standard OR (95% CI)	*p* value	standard OR (95% CI)	*p* value
MPP-CV	(per 4.52%)		(per 4.61%)	
Model 1	1.13 (1.08, 1.18)	<.001	1.10 (1.04, 1.16)	<.001
Model 2	1.12 (1.07, 1.18)	<.001	1.08 (1.03, 1.14)	.003
Model 3	1.09 (1.04, 1.15)	<.001	1.08 (1.02, 1.14)	.004
Model 4	1.10 (1.04, 1.15)	<.001	1.08 (1.02, 1.14)	.01
Model 5	1.08 (1.02, 1.13)	.003	1.06 (1.00, 1.12)	.05
MPP-VIM	(per 0.073units)		(per 0.28 units)	
Model 1	1.09 (1.04, 1.14)	<.001	1.09 (1.04, 1.15)	<.001
Model 2	1.08 (1.03, 1.14)	<.001	1.07 (1.02, 1.13)	.007
Model 3	1.06 (1.01, 1.11)	.012	1.08 (1.02, 1.13)	.007
Model 4	1.07 (1.02, 1.12)	.007	1.06 (1.00, 1.12)	.05
Model 5	1.07 (1.02, 1.12)	.005	1.06 (1.00, 1.12)	.04

Model 1: univariate analyses; model 2: model 1 plus adjusting age, gender, body mass index, ethnicity; model 3: model 2 plus adjusting surgery admission, cardiovascular ICU, history of hypertension, history of diabetes, history of chronic kidney disease, history of chronic heart failure, modified SOFA score and OASIS during the first 24 h; model 4: model 3 plus adjusting cumulative vasopressor dose during the first 24 h, sedatives use or not during the first 24 h, mechanical ventilation or not during the first 24 h, transfusion of RBCs during the first 24 h, AKI at the first 24 h, sepsis or not before the 24 h of ICU admission, exposure to ≥ 2 nephrotoxins before and after 24 h of ICU admission; model 5: additionally adjusting time-weighted average MPP based on model 4.

### Sensitivity and subgroup analyses

The relationship between primary endpoint and MPP-VIM was similar to that of MPP-CV (Supplementary Figure 2). In univariate and multivariate logistic regression models, when MPP-VIM increased per 1-SD, which was 0.073 units in eICU-CRD and 0.28 units in MIMIC-IV, respectively, the fully adjusted sOR in model 5 for deterioration of renal function was 1.07 (95% CI, 1.02–1.12, *p* = .005) and 1.06 (95% CI, 1.00–1.12, *p* = .04) (Table 2). The results were robust when baseline creatinine was defined by the nadir creatine during 7 days before and after ICU admission (Supplementary Figure 3, Supplementary Table 3).

**Figure 3.**
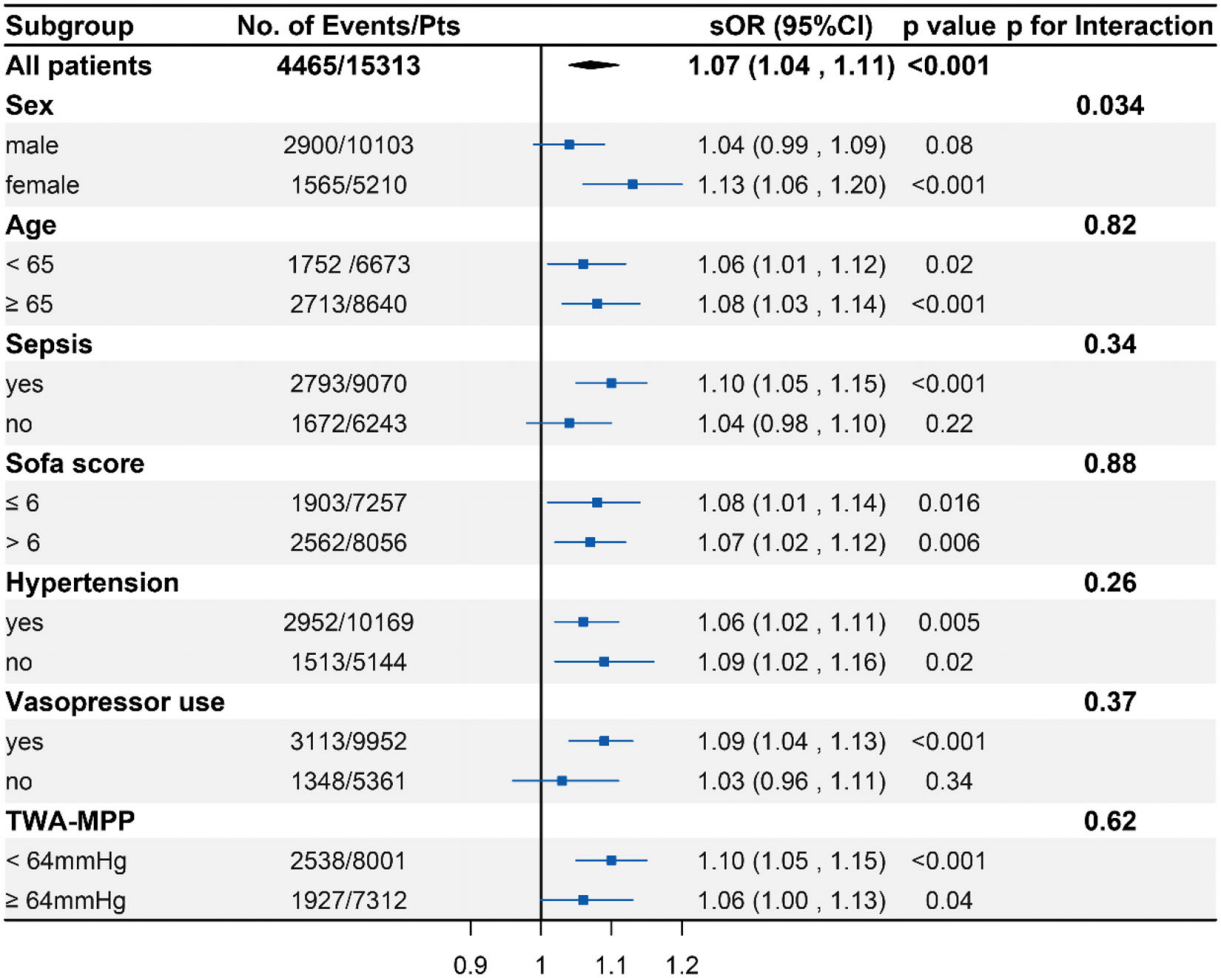
Adjusted odds ratios and 95% CIs for subsequent deterioration of renal function associated with the increased MPP-CV in different subgroups.

Subgroup analyses were conducted across the pooled population of the two databases who were male or female, elderly (age ≥65 years) or not, with or without hypertension, sepsis, higher than median SOFA score on the first day of ICU admission or not, vasopressor use or not and higher than median TWA-MPP or not. The main results were displayed in Figure 3. Female patients were more likely to experience subsequent deterioration of renal function due to high MPP-CV. And in the pooled analyses of the two databases including cardiac surgery, medical sepsis and others (Table 3, supplementary Table 4), the results showed heterogeneity in patients with medical sepsis (sOR = 1.11, 95% CI, 1.01–1.21, *p* = .02), cardiac surgery (sOR = 1.04, 95% CI, 1.00–1.10, *p* = .08) and others (sOR = 1.10, 95% CI, 1.03–1.18, *p* = .007).

**Table 3.** Pooled analysis of the association of MPP-CV and subsequent deterioration of renal function in different types of patients.

Models for subsequent deterioration of renal function	Medical sepsis (*n* = 2506)		Cardiac surgery (*n* = 8385)		Others (*n* = 4422)	
	Standard OR (95% CI)	*p* value	Standard OR (95% CI)	*p* value	Standard OR (95% CI)	*p* value
MPP-CV	(per 4.68%)		(per 4.10%)		(per 5.31%)	
Model 1	1.16 (1.06, 1.26)	<.001	1.09 (1.04, 1.14)	<.001	1.16 (1.08, 1.23)	<.001
Model 2	1.16 (1.07, 1.26)	<.001	1.06 (1.01, 1.11)	.02	1.15 (1.08, 1.23)	<.001
Model 3	1.13 (1.04, 1.23)	.004	1.05 (1.00, 1.10)	.03	1.12 (1.05, 1.20)	<.001
Model 4	1.11 (1.01, 1.21)	.02	1.07 (1.02, 1.12)	.006	1.12 (1.05, 1.20)	<.001
Model 5	1.11 (1.01, 1.21)	.02	1.04 (1.00, 1.10)	.08	1.10 (1.03, 1.18)	.007

Model 1: univariate analyses; model 2: model 1 plus adjusting age, gender, body mass index, ethnicity; model 3: model 2 plus adjusting surgery admission, cardiovascular ICU, history of hypertension, history of diabetes, history of chronic kidney disease, history of chronic heart failure, modified SOFA score and OASIS during the first 24 h; model 4: model 3 plus adjusting cumulative vasopressor dose during the first 24 h, sedatives use or not during the first 24 h, mechanical ventilation or not during the first 24 h, transfusion of RBCs during the first 24 h, AKI at the first 24 h, sepsis or not before the 24 h of ICU admission, exposure to ≥ 2 nephrotoxins before and after 24 h of ICU admission; model 5: additionally adjusting time-weighted average MPP based on model 4.

## Discussion

This multicenter *post hoc* analysis demonstrated that increased MPP-CV in the first 24 h after ICU admission was associated with deterioration of renal function in subsequent 48 h. This association remained significant after adjustment for potential confounding factors, such as baseline demographic data, comorbidities, admission illness severity scores, risk factors for AKI and TWA-MPP. Additionally, the association may be heterogeneous in patients with cardiac surgery, medical sepsis and others.

The BP is always in a state of dynamic balance, regulating various external stimuli and maintaining dynamic balance. This leads to the concept of BPV, which mainly reflects the efficiency of cardiovascular control mechanisms in regulating BP, including central neural and humoral rhythms and responses to environmental disturbances [30]. In previous studies, the relationship of increased short-term or long-term BPV and adverse renal outcomes have been confirmed in different populations. In patients with stable coronary heart disease, higher BPV during hospitalization was associated with decreased renal function [31]. Two studies about intraoperative BPV also confirmed that a higher BPV was linked with postoperative AKI and postoperative mortality in noncardiac surgery [29,32]. Short-term BPV is independently associated with early renal abnormalities in essential hypertension as well [33]. Nevertheless, there is little data on BPV and deterioration of renal function in critically ill patients [15]. The results of our study showed for the first time of the relationship of MPPV and subsequent deterioration of renal function in critically ill patients. Moreover, we confirmed the results in the patients with medical sepsis, cardiac surgery and others.

When the body’s BP fluctuates, the kidney maintains its own perfusion mainly through two mechanisms which are the myogenic response and the tubuloglomerular feedback response. However, this classic curve of automatic blood flow regulation was obtained from the experiment, which gradually reduces the renal perfusion pressure. Therefore, this description is valid only for very slow changes in BP and the effect of faster BP fluctuations on renal blood flow cannot be correctly described. Critically ill patients are known for high incidence of anxiety [34], delirium [35], sleep loss [36], abnormal central and autonomic nervous regulation [37]. Under such situations, patients tend to have difficulty maintaining stable renal perfusion, then exhibiting deterioration of subsequent renal function. Further physiological studies are required to reveal the potential mechanism between increased MPPV and deterioration of renal function.

Additionally, the association between MPPV and deterioration of renal function may be heterogeneous in patients with cardiac surgery, medical sepsis and others. Critically ill patients with medical sepsis are characterized with endothelial dysfunction [38]. Thus, the same rise and fall in MPP may lead to greater fluctuation of renal blood flow compared to those diseases without marked endothelial dysfunction. And for patients undergoing cardiac surgery, they presented with decreased perfusion pressure characterized by renal venous congestion [39]. The correlation between absolute level of MPP and the deterioration of renal function may be more significant [40]. After adjusting for TWA-MPP, the correlation between MPPV and the deterioration of renal function weakened. In patients after abdominal surgery, intra-abdominal pressure may have a greater effect on renal perfusion [41]. However, our study excluded patients with acute compartment syndrome, and the number of patients undergoing abdominal surgery was limited, so they were not discussed separately.

Our subgroup analyses revealed heterogeneity in the association between MPPV and deterioration of subsequent renal function in patients of different genders.Why female patients were more likely to experience subsequent deterioration of renal function due to high MPP-CV was unknown and merited further studies.

The population in this study was limited to those with CVP monitoring in ICU. Compared with a standard ICU population [42], a large proportion of the study population (36% in standard ICU vs >70% in two databases) was admitted to hospital due to surgery and most patients were admitted to cardiovascular ICU (53.6% and 91.0% in two databases). Proportion of patients with the history of chronic heart failure (9.1% in standard ICU vs 16.7% and 24.7% in two databases) and diagnosed with sepsis (18% in standard ICU vs 37.3% and 87.2% in two databases) was also higher than that in the standard ICU.

This study has several limitations. Firstly, the included patients were not representative of typical ICU patients, as they received continuous monitoring of CVP. Therefore, the results may not be fully applied to the normal ICU population. Secondly, it was hard to prove the causal relationship between MPPV and the primary endpoint as the study was observational, despite using two databases to confirm the association. The question of whether MPPV was a marker of severity of illness or a potential target to improve prognosis required randomized trials to answer. Thirdly, our study did not account for advanced hemodynamic data such as cardiac index, peripheral vascular resistance and mechanical ventilation parameters like positive end-expiratory pressure. Fourth, although we used multiple risk adjustments and included many potential confounders, some residual confounders may be responsible for the observed association. Despite these limitations, this was the first clinical investigation to explore the relationship between MPPV and subsequent deterioration of renal function in critically ill patients. It provided preliminary evidence to support maintaining stable MPP in hemodynamic management in critically ill patients with CVP monitoring.

## Conclusion

Increased MPPV was associated with an increased risk of subsequent deterioration of renal function in critically ill patients with CVP monitoring. Maintaining stable MPP may reduce the risk of deterioration of renal function.

## Supplementary Material

Supplemental Material
