# Supplementary material for: Increased mean perfusion pressure variability is associated with subsequent deterioration of renal function in critically ill patients with central venous pressure monitoring: a retrospective observational study
Source: Ren Fail. 2022 Nov 11;44(1):1986–94. doi: 10.1080/0886022X.2022.2120822 (PMC9662057; doi:10.1080/0886022X.2022.2120822)
Supplement: Supplemental Material [file IRNF_A_2120822_SM1665.pdf]

Supplementary methods

Nephrotoxins: It was defined as vancomycin, non-steroidal anti-inflammatory drugs, amphotericin, aminoglycosides, angiotensin inhibitors or angiotensin receptor blockers, calcineurin inhibitors in this research.

Sedatives: It was defined as pentobarbital, atracurium, dexmedetomidine, diazepam, midazolam, propofol, lorazepam, haloperidol in the three databases.

Modified SOFA: It was defined as the SOFA score minus cardiovascular component because that the multivariate modes had taken MPP or MAP and vasopressor dose into account.

Norepinephrine Equivalents: All vasopressors were standardized according to the following conversion scale below.

**Drug**	**Dose**	**Norepinephrine Equivalent**
Epinephrine	0.1 mcg/kg/min	0.1 mcg/kg/min
Norepinephrine	0.1 mcg/kg/min	0.1 mcg/kg/min
Dopamine	10 mcg/kg/min	0.1 mcg/kg/min
Dobutamine	10 mcg/kg/min	0.1 mcg/kg/min
Milrinone	1.0 mcg/kg/min	0.1 mcg/kg/min
Phenylephrine	1.0 mcg/kg/min	0.1 mcg/kg/min
Vasopressin	0.001U/kg/min	0.1 mcg/kg/min

Reference. Belletti A, Lerose CC, Zangrillo A, Landoni G. Vasoactive-Inotropic Score: Evolution, Clinical Utility, and Pitfalls [published online ahead of print, 2020 Sep 22]. J Cardiothorac Vasc Anesth. 2020;S1053-0770(20)31035-1.

Cardiovascular ICU: defined as “Cardiac ICU” or “CCU-CTICU” or “CSICU”

or “CTICU” in eICU-CRD, and “Cardiac Vascular Intensive Care Unit (CVICU)” or “Coronary Care Unit (CCU)” in MIMIC-IV database, respectively.

Supplementary tables

Supplementary Table 1. Calculation Formula of variability parameters.

**Parameter**	**Formula**
Coefficient of variation (CV)	$\frac{SD}{\bar{x}}$
Variation independent of mean (VIM)	k× $\frac{SD}{\bar{x}^b}$

Note: n is the number of MPP readings, $\bar{x}$ is the mean value and w refers to the time of each interval. For VIM, linear regression fitting log (SD) with log (x) was performed. The “k” was the exponential of β0 and the “b” was the β1 of the linear regression model.

Supplementary Table 2. Baseline characteristics and outcomes of the study population.

**Variables**	**eICU-CRD (N=8590)**	**MIMIC-IV (N=6723)**
Year of data	2014-2015	2008-2019
Hospital size		
< 100 beds	66 (0.8)	0 (0.0)
100 – 249 beds	1092 (12.7)	0 (0.0)
250 – 499 beds	2190 (25.5)	0 (0.0)
≥ 500 beds	4060 (47.3)	6723 (100.0)
Not available	1183 (13.7)	0 (0.0)
Teaching hospital	3060 (35.6)	6723 (100.0)
Region		
Midwest	2857 (33.3)	0 (0.0)
Northeast	521 (6.1)	6723 (100.0)
South	2493 (29.0)	0 (0.0)
West	2115 (24.6)	0 (0.0)
Not available	604 (7.0)	0 (0.0)
Mechanical ventilation (%)	6416 (74.7)	6519 (97.0
Transfusion of RBCs (%)	474 (5.5)	1696 (25.2)
Transfusion of RBCs (mL)	0 (0, 0)	0 (0, 275)
Vasopressor usage (%)	4534 (52.8)	5418 (80.6)
Vasopressor dose (NE, mg)	0.00 (0.00, 2.25)	1.08 (0.08, 4.90)
Sepsis (%)	3203 (37.3)	5867 (87.3)
Exposure to ≥ 2 nephrotoxins (%)	1503 (17.5)	1993 (29.6)
Vancomycin	1824 (21.2)	3528 (52.5)
NSAIDs	4546 (52.9)	4481 (66.7)
Amphotericin	0 (0.0)	1 (0.0)
Aminoglycosides	0 (0.0)	59 (0.9)
ACEIs or ARBs	405 (4.7)	258 (3.8)
Calcineurin inhibitors	31 (0.4)	96 (1.4)
AKI in the first 24 hours (%)	4011 (46.7)	4417 (65.7)
Stage 1	1446 (16.8)	1670 (24.8)
Stage 2	1702 (19.8)	2378 (35.4)
Stage 3	863 (10.0)	369 (5.5)
Modified SOFA Score	6 (4, 8)	4 (3, 6)
OASIS Score	30(24, 36)	34 (30, 39)
MPP data		
Measurement times of MPP	240 (201, 274)	24 (20, 28)
Durations of MPP (hours)	20.0 (17.0, 23.0)	18.4 (16.3, 20.2)
Max measurement interval (minutes)	20 (17, 23)	60 (60, 120)
Exposure of interest		

MPP-CV (%)	11.7 (9.4, 14.6)	12.6 (10.2, 15.5)
MPP-VIM (units)	0.20 (0.16, 0.24)	0.81 (0.66, 0.99)
Outcomes		
Deterioration of renal function in the next 48 hours (%)	2437 (28.4)	2028 (30.2)
AKI in the next 48 hours (%)	4486 (52.2)	4726 (70.3)
Stage 1	1312 (15.3)	2050 (30.5)
Stage 2	1773 (20.6)	2115 (31.5)
Stage 3	1401 (16.3)	561 (8.3)
In-hospital mortality (%)	785 (9.1)	221 (3.3)
Hospital length of stay (days)	8.4 (5.7, 13.9)	5.3 (4.3, 8.1)
ICU mortality (%)	550 (6.4)	174 (2.6)
ICU length of stay (days)	2.9 (1.9, 5.0)	2.1 (1.3, 3.7)

Continuous variables were expressed as median (interquartile range) as the distributions are skewed, and categorical variables were expressed as number (percentage).

ACEI: Angiotensin inhibitors; AKI: acute kidney injury; ARBs: Angiotensin receptor blockers; ICU: intensive care unit; MAP: mean arterial pressure; MPP: mean perfusion pressure; NE: norepinephrine equivalents; NSAIDs: Non-steroidal anti-inflammatory drugs; OASIS: Oxford Acute Severity of Illness Score; RBC: red blood cell; SOFA: Sequential Organ Failure Assessment; TWA: time weighted-average.

Supplementary Table 3. Association of MPP-CV and subsequent deterioration of renal function in different cohorts when baseline creatinine was determined by the nadir creatine during 7 days before and after ICU admission.

Models for subsequent deterioration of renal function	eICU-CRD		MIMIC-IV	
	sOR / adjusted sOR (95% CI)	*p* value	sOR / adjusted sOR (95% CI)	*p* value
	(per 4.52%)		(per 4.61%)	
Univariable Model	1.14 (1.09,1.19)	<0.001	1.10 (1.05,1.16)	<0.001
Multivariable Model 1	1.13 (1.08,1.18)	<0.001	1.08 (1.03,1.14)	0.002
Multivariable Model 2	1.09 (1.04,1.14)	<0.001	1.08 (1.03,1.14)	<0.001
Multivariable Model 3	1.09 (1.04,1.14)	<0.001	1.08 (1.02,1.14)	0.009
Multivariable Model 4	1.06 (1.02,1.12)	0.01	1.06 (1.01,1.13)	0.03

Model 1: univariate analyses; model 2: model 1 plus adjusting age, gender, body mass index, ethnicity; model 3: model 2 plus adjusting surgery admission, cardiovascular ICU, history of hypertension, history of diabetes, history of chronic kidney disease, history of chronic heart failure, modified SOFA score and OASIS during the first 24 hours; model 4: model 3 plus adjusting cumulative vasopressor dose during the first 24 hours, sedatives use or not during the first 24 hours, mechanical ventilation or not during the first 24 hours, transfusion of RBCs during the first 24 hours, AKI at the first 24 hours, sepsis or not before the 24 hours of ICU admission, exposure to ≥ 2 nephrotoxins before and after 24 hours of ICU admission; model 5: additionally adjusting time-weighted average MPP based on model 4.

Supplementary Table 4. Baseline characteristics of the pooled cohort in patients with medical sepsis, cardiac surgery and others.

**Pooled cohort**	Medical Sepsis	Cardiac Surgery	Others	P value
n	2506	8385	4422	
Age (year)	66.00 (58.00, 76.00)	68.00 (60.00, 76.00)	66.00 (56.00, 75.00)	<0.001
Male (%)	1655 (66.0)	5767 (68.8)	2681 (60.6)	<0.001
Body mass index (kg/m^2)	28.41 (24.72, 32.82)	28.55 (25.10, 32.76)	28.05 (24.39, 32.81)	<0.001
White race (%)	1879 (75.0)	6593 (78.6)	3223 (72.9)	<0.001
Surgical admission (%)	0 (0.0)	8385 (100.0)	3190 (72.1)	<0.001
Cardiovascular ICU (%)	1470 (58.7)	6812 (81.2)	2445 (55.3)	<0.001
History of hypertension (%)	1553 (62.0)	5890 (70.2)	2726 (61.6)	<0.001
History of diabetes (%)	646 (25.8)	2474 (29.5)	1111 (25.1)	<0.001
History of chronic kidney disease (%)	385 (15.4)	1074 (12.8)	646 (14.6)	0.001
History of chronic heart failure (%)	576 (23.0)	1679 (20.0)	846 (19.1)	<0.001
Vasopressor (%)	1893 (75.5)	5808 (69.3)	2251 (50.9)	<0.001
Vasopressor dose (NE, mg)	1.72 (0.00, 8.35)	0.32 (0.00, 3.30)	0.00 (0.00, 1.84)	<0.001
Transfusion of RBCs (%)	468 (18.7)	1222 (14.6)	480 (10.9)	<0.001
Sedatives use (%)	1817 (72.5)	5165 (61.6)	2190 (49.5)	<0.001
Mechanical ventilation (%)	2222 (88.7)	7580 (90.4)	3133 (70.9)	<0.001
Transfusion of RBCs (mL)	0.00 (0.00, 0.00)	0.00 (0.00, 0.00)	0.00 (0.00, 0.00)	<0.001
Sepsis (%)	2506 (100.0)	5078 (60.6)	1486 (33.6)	<0.001
Exposure to ≥ 2 nephrotoxins (%)	946 (37.7)	1983 (23.6)	567 (12.8)	<0.001
AKI in the first 24 hours (%)	1753 (70.0)	4231 (50.5)	2444 (55.3)	<0.001
Deterioration of renal function in the next 48 hours (%)	775 (30.9)	2540 (30.3)	1150 (26.0)	<0.001
Modified SOFA Score	6 (4, 8)	5 (3, 7)	5 (3, 8)	<0.001
OASIS	35 (30, 41)	31 (26, 36)	31 (24, 38)	<0.001
TWA-CVP (mmHg)	10.38 (7.58, 13.64)	10.13 (7.72, 12.75)	9.75 (6.93, 12.86)	<0.001
TWA-MPP (mmHg)	63.10 (58.11, 69.30)	62.85 (58.30, 67.88)	65.36 (59.69, 72.41)	<0.001
TWA-MAP (mmHg)	73.78 (69.57, 78.79)	73.22 (69.10, 77.47)	75.43 (70.39, 81.52)	<0.001
MPP-CV (%)	12.39 (9.97, 15.71)	11.98 (9.77, 14.73)	11.99 (9.51, 15.17)	<0.001

Supplementary figures

**Pre-ICU LOS (hours)**	**<0.17**	**0.17-4.94**	**4.95-24.00**	**24.01-311.80**	**>311.80**	
Score	5	3	0	2	1	
**Age (years)**	**<24**	**24-53**	**54-77**	**78-89**	**>90**	
Score	0	3	6	9	7	
**GCS**	**15**	**14**	**8-13**	**3-7**		
Score	0	3	4	10		
**Heart Rate (/min)**	**<33**	**33-88**	**89-106**	**107-125**	**>125**	
Score	4	0	1	3	6	
**MAP (mmHg)**	**<20.65**	**20.65-50.99**	**51-61.32**	**61.33-143.44**	**>143.44**	
Score	4	3	2	0	3	
**Respiratory Rate (/min)**	**<6**	**6-12**	**13-22**	**23-30**	**31-44**	**>44**
Score	10	1	0	1	6	9
**Temperature (℃)**	**<33.22**	**33.22-35.93**	**35.94-36.39**	**36.40-36.88**	**36.89-39.88**	**>39.88**
Score	3	4	2	0	2	6
**Urine Output（cc/day)**	**<671**	**671-1426.99**	**1427-2543.99**	**2544-6896**	**>6896**	
Score	10	5	1	0	8	
**Ventilated**	**No**	**Yes**				
Score	0	9				
**Elective Surgery**	**No**	**Yes**				
Score	6	0				

Supplementary Figure1. The Oxford Acute Severity of Illness Score (OASIS). The final OASIS score is the sum of all the component weights.

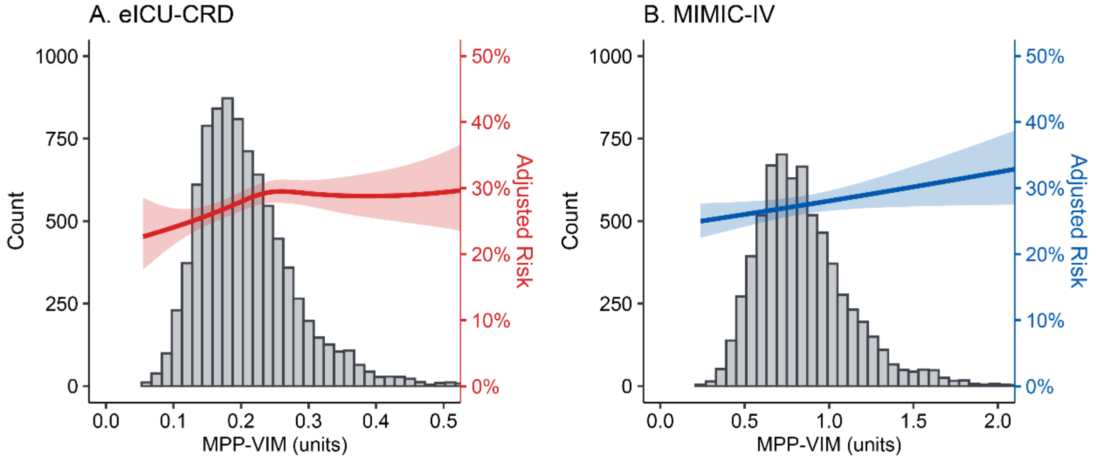

Supplementary Figure2. The association between MPP-VIM and subsequent deterioration of renal function fitted by general additive models and the histograms of MPP-VIM in two databases.

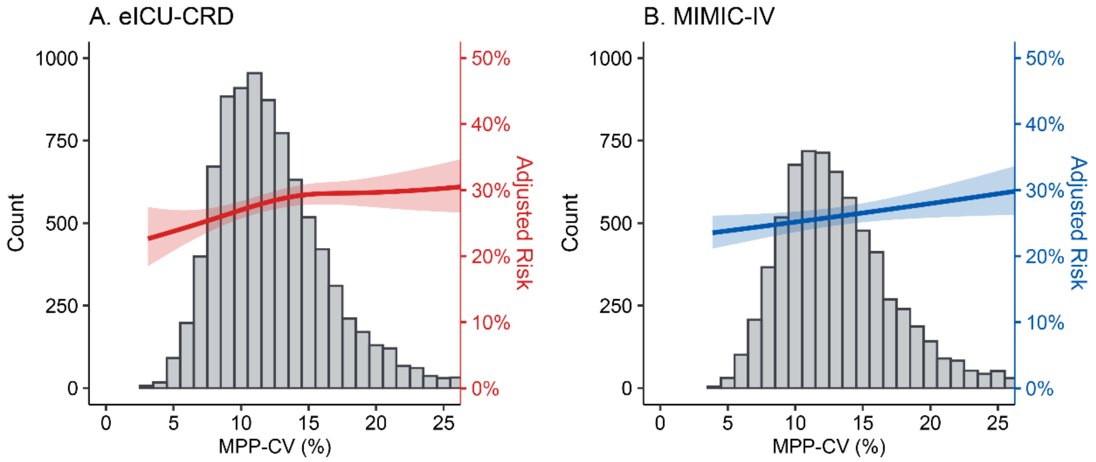

Supplementary Figure3. The association between MPP-CV and subsequent deterioration of renal function fitted by general additive models when baseline creatinine was determined by the nadir creatine during 7 days before and after ICU admission and the histograms of MPP-CV in two databases.
